# GAN-SAE based fault diagnosis method for electrically driven feed pumps

**DOI:** 10.1371/journal.pone.0239070

**Published:** 2020-10-22

**Authors:** Hui Han, Lina Hao, Dequan Cheng, He Xu

**Affiliations:** 1 School of Mechanical Engineering and Automation, Northeastern University, Shenyang, China; 2 School of Mechanical Engineering, Shenyang Ligong University, Shenyang, China; National Huaqiao University, CHINA

## Abstract

The running of high-speed electrically driven feed pump has a direct impact on the safety of personnel equipment and economic benefits of power plant, as the result, intelligent condition monitoring and fault diagnosis of electrically driven feed pump becomes an urgent need. In the practical process of electrically driven feed pump fault diagnosis, the running of the equipment is in normal state for a long time, occasionally, with faults, which makes the fault data very rare in a large number of monitoring data, and makes it difficult to extract the internal fault features behind the original time series data, When the deep learning theory is used in practice, the imbalance between the fault data and the normal data occurs in the operation data set. In order to solve the problem of data imbalance, this paper proposes a fault diagnosis method of GAN-SAE. This method first makes compensation for the imbalance of sample data based on the Generative Adversarial Network (GAN), and then uses the Stacked Auto Encoder (SAE) method to extract the signal features. By designing the fault diagnosis program, compared with only using SAE, back propagation neural networks (BP) and multi-hidden layer neural networks(MNN) method, the GAN-SAE method can offer better capability of extracting features, and the accuracy of fault diagnosis of electrically driven feed pump could be improved to 98.89%.

## Introduction

High-speed electrically driven feed pump is an essential equipment of utility boiler, which plays a very important role in the normal running and safety of power utility. However, compared with large-scale equipment such as boiler, steam turbine and generator, electrically driven feed pump unit has not been given enough attention, which is the weak link of equipment condition monitoring as well as the major reasons for the unexpected shutdown of the generator set **[1].** So it is necessary to monitor and diagnose running condition of electrically driven feed pump unit. At present, due to many monitoring points, high sampling rate, long time of monitoring data collection and other reasons, the electrically driven feed pump monitoring system obtains a large amount of health monitoring data, which on the one hand promotes the field of equipment health monitoring into the "big data era", on the other hand, it also puts forward higher requirements for fault information mining and diagnosis **[2].** Taking the electrically driven feed pump of power utility as an example, although there is a large amount of monitoring data, when the mechanical failures occurring occasionally, there is a small amount of fault state data, which presents the imbalance between fault data and normal data, which makes it very difficult to diagnose the fault based on data-driven methods.

Aiming at the problem of imbalanced data sample, most of the research is carried out from the data and algorithm level [3–5]. From the data level, the training set is usually reconstructed, mainly by oversampling the minority samples and undersampling the majority samples. The most typical is the synthetic minority oversampling technique method (SMOTE) [6], which increases the number of minority samples by randomly interpolating virtual samples to achieve a balanced sample data set. Mao et al. [7] introduced the master curve to extract the characteristics of the flow distribution, and used the SMOTE algorithm to oversample and undersample the minority and majority samples to balance the fault sample data. Finally, the online sequence extreme learning machine (OSELM) classifier was used for bearing fault diagnosis. Tao Xinmin et al. [8] used the SMOTE algorithm to generate minority samples, then used the original samples and newly generated samples to train SVM, and realized bearing fault diagnosis. E. Ramentol et al. [9] combined fuzzy rough set theory with SMOTE, and used two different thresholds to process the minority class and the majority class respectively to achieve a balanced data set, and finally used the C4.5 classifier to perform the fault of the high-voltage circuit breaker diagnosis. From the perspective of algorithm, it mainly improves the diagnostic performance by improving the classification algorithm, including cost-sensitive learning method and integrated learning method, etc. The cost-sensitive learning method mainly includes CS decision trees and neural networks, and integrated learning method includes Bagging algorithm and Adaboost algorithm. Duan et al. [10] studied a support vector data description method based on cross-tree. This method drew a binary tree structure of multiple clusters in the order of the distance from the cluster to the cluster calculated by the Mahalanobis distance. Then used the PSO algorithm to optimize the parameters of the support vector data description (SVDD), and finally used the binary tree network to complete the fault diagnosis of the rotor in the rotor system under the imbalanced sample size. Fan et al. [11] proposed an entropy-based fuzzy support vector machine (EFSVM), which guarantees the importance of the minority class by assigning relatively large fuzzy memberships to the minority class, thereby realizing various imbalanced sample set patterns recognition. Guo et al. [12] first used BPSO for feature selection, then used the Adaboost method to train multiple k-NN classifiers, and finally performed pattern recognition under various imbalanced sample sets. Currently, researchers apply the deep neural network learning method to the problem of sample imbalance in fault diagnosis. Lei et al. [13] studied a classifier loss strategy with adjustable weight coefficients combined with a deep normalized convolutional neural network (DNCNN). In this method, a small number of samples were given a large weight coefficient according to the degree of imbalance, so as to complete the fault diagnosis of bearings. Zhang et al. [14] designed a weighted minor-class oversampling self-coding network (WMODAE), in which the weighted minor-class oversampling technique was used to obtain the balanced sample data set, then the effective features were extracted with an automatic encoder, and finally the bearing fault diagnosis was realized with decision tree C4.5 algorithm.

Although the above-mentioned traditional imbalanced sample processing methods have improved the classification accuracy of imbalanced samples to a certain extent, they still have several shortcomings: (1) Virtual sample generation methods such as SMOTE are prone to generate noisy samples or invalid samples, especially in the case of "non-convex" fault sample space and large change conditions. (2) The classification performance of the existing classification network is reduced under the data set after sample expansion. (3) When the sample size imbalance ratio between the majority class and the minority class is too large, the effect of a large number of improved algorithms will become smaller and smaller.

Generative adversarial networks (GAN) is a new unsupervised learning model, which was first proposed by Goodfellow etc in 2014 [15]. GAN is widely used to solve the problem of data imbalance because of its powerful data generation capability. Ref. [16] used GAN generator to capture the real distribution of the original samples, and then generated new samples with similar distribution to the original samples, expanded the number of training fault samples, and then improved the accuracy of planetary gearbox fault diagnosis. Ref. [17] proposed a new method of selective GAN, solving the problem of high data imbalance in the fault diagnosis of reciprocating machinery. Ref. [18] proposed a deep convolutional GAN (DCGAN) model to simulate the original distribution from minority classes and generate new data to solve the data imbalance problem, improving the accuracy of fault diagnosis. In addition to the above studies, there are many studies around deep learning methods such as variational auto encoders (VAE) [19] and auto encoders (AE) [20], combined with the advantages of generative adversarial networks, aiming to improve the accuracy and authenticity of samples generated by GAN. For the research of pump fault diagnosis, there is no relevant literature on the application of the GAN method.

Therefore, in order to the problem of data sample imbalance in the fault diagnosis of electrically driven feed pump, this paper combines the powerful data generation capabilities of the GAN and deep learning technology of the stacked auto encoder (SAE) with feature learning and extraction capabilities [21–23], proposes a fault diagnosis method of GAN-SAE for the first time. This method firstly makes compensation for the imbalance of sample data based on the GAN, uses the SAE method to extract the signal features, integrates GAN with SAE networks to design the fault diagnosis program, and applies it in the fault diagnosis system of the electrically driven feed pump, so as to improve the diagnosis accuracy. This paper mainly consists of four parts. Firstly, it introduces the related theoretical basis. Secondly, it focuses on the GAN-SAE fault diagnosis algorithm. Thirdly, it gives the fault diagnosis experiment and analysis. Finally, it draws the conclusion and analysis.

## 1 Introduction to GAN and SAE

### 1.1 Generative adversarial networks (GAN)

**Fig 1:**
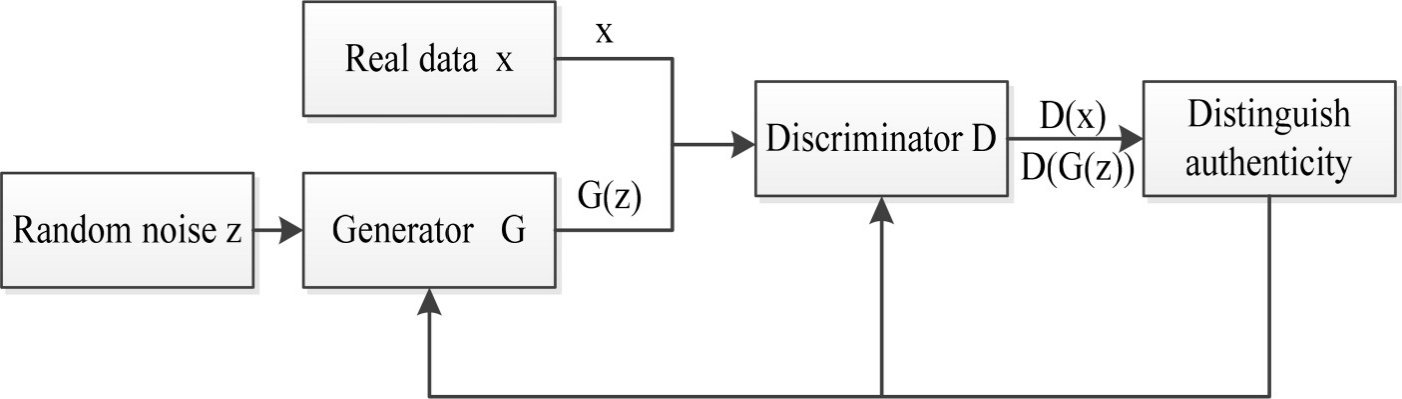
General GAN structure diagram.

Generative adversarial network is a new type of deep learning network model, which directly generate similar distributions from sample data. In Ref. [13], this network model is structurally inspired by the minimax two-player game theory, and in game theory, this model is composed of a generator (G) and a discriminator (D). The generator captures the potential distribution of real data samples and generates new data samples; the discriminator is a binary classifier, its objective is to determine whether the input sample is real or generated by the generator. Therefore in the training process of network model, the minimax two-player game composed by generator and discriminator. The goal of the training is to get the game to Nash Equilibrium [24]. The general structure of GAN is shown in Fig 1. *G*(*z*) represents the generated sample output by the generator, and *D*(*G*(*z*)) represents the discriminator's evaluation value of the generated sample. *x* is the real sample and *D*(•) is the discriminator output.

In the network model, the generator G receives a set of random noise z as input, and outputs a generated sample G (z). The input of the discriminator D includes the real samples x from the real data distribution and the fake samples G (z) generated by the generator G. The output is a probability value from 0 to 1. The value represents that the probability which discriminator D considers the input data to be real. At this time, the discriminator D is equivalent to a two-classifier **to determine** whether the input data comes from the real data distribution or the generated data distribution [25]. For example, when D considers the input data to be the real sample x, it outputs 1; otherwise, when D considers the input data to be the samples G (z), it outputs 0.

During the training process, the discriminator D discriminates the fake samples generated by the generator G, and G updates its network parameters according to the discriminant results of D, and continuously improves the generation ability, so that the generated samples gradually approach the real data distribution, thereby they can cheat D. In order not to be deceived by G, D tries to find the difference between the real sample and the fake sample generated by G, and continuously improves its discriminate ability. Specifically, for the fake sample, the goal of the discriminator D is to make the output close to 0, and the goal of the generator G is to make it close to 1. The generator G and the discriminator D continuously optimize the respective networks through their iterative training process until they reach a balanced state.

The design of the generative adversarial network loss function is based on the Nash equilibrium of the two-player game. The calculation formula of the loss function of the generator used in this paper is shown in Eq (1).

G_loss=log(D(G(z))) (1)

The goal of the generator parameter update is to make *D*(*G*(*z*)) approach 1, that is, the discriminator will judge the generated sample as a true sample, and take the logarithm of the value as the loss function, so that the target can be converted to make the loss function approach 0.

The calculation formula of the discriminator's loss function is shown in Eq (2).

D_loss=−log(D(x))+log(1−D(G(z))) (2)

The purpose of the discriminator parameter update is to make the evaluation value of the real sample approach 1, and the evaluation value of the generated sample tend to 0, which maximizes the discrimination ability between the generated sample and the real sample. By taking the logarithm of *D*(*x*) and 1−*D*(*G*(*z*)), and summing them as the discriminator's loss function, the goal can be converted to make the loss function approach zero. This function embodies a major characteristic of GAN, that is, the minimax two-player game is formed by the loss function of the generator and discriminator, and the convergence of the network is promoted through the process of the game. At the same time, due to the existence of Nash equilibrium in game theory, network convergence is guaranteed and network reliability is improved.

### 1.2 Stacked Auto Encoder (SAE)

**Fig 2:**
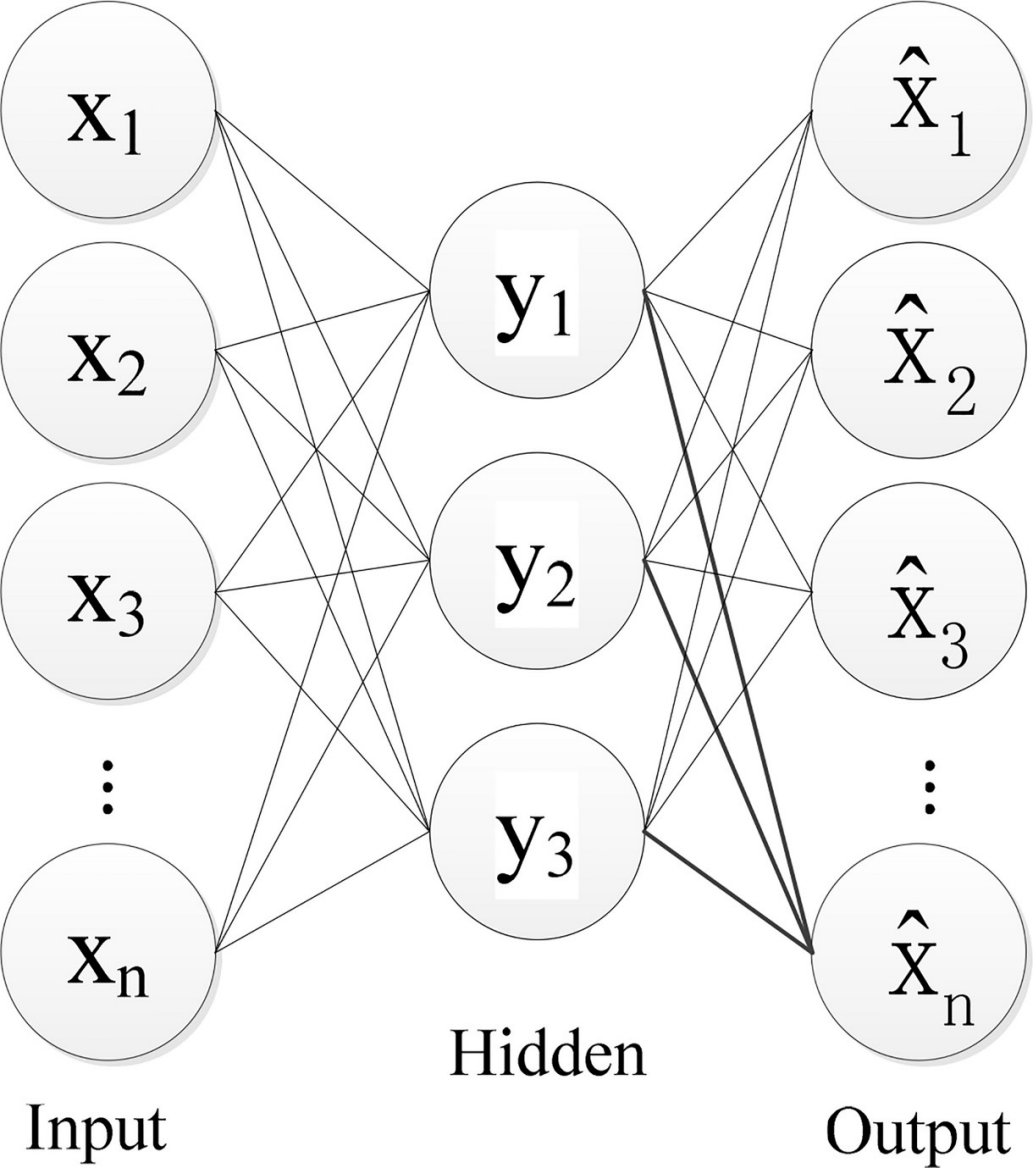
Schematic diagram of AE structure.

The Auto Encoder (AE) is a typical single hidden layer neural network proposed by Rumelhart in Ref. [26] 1986. Since this network keeps the input and output as consistent as possible. In this case, the hidden layer feature extraction and parameter learning are implemented in an unsupervised manner. Then it's called AE. In the structure of AE neurons possess the same amount of data in the input and output layers. The process from input to hidden layer is called encoding, and that from hidden layer to output is called decoding respectively. The schematic diagram of the Auto Encoder network structure is shown in Fig 2.

Each neuron of Auto Encoder network uses a fully connected method to weight the sum of the input vector *X* = (*x*_1_, *x*_2_,⋯, *x*_*n*_), then add the bias and go through the activation function to obtain the encoding vector *Y* = (y_1_, y_2_,⋯, y_m_) of the hidden layer. The encoding vector is remapped to the output vector $\hat{X}={\hat{x}}_{1},\;{\hat{x}}_{2},\cdots ,\;{\hat{x}}_{n})$ through the decoding network. Finally, it is necessary to ensure that the input and output layers are as similar as possible, that is, the weights and offsets should be adjusted and optimized with the goal of minimizing the reconstruction error. The mathematical expression of the Auto Encoder network is as follows:

$$Y=\sigma_{a}(W_{a}•X+b_{a})$$ (3)

$$\hat{X}=\sigma_{s}(W_{s}•Y+b_{s})$$ (4)

$$\mathrm{minL}\;(\theta )={\left\|\hat{X}‐X\right\|}_{2}^{2}$$ (5)

$$\theta =\left[W_{a},\;b_{a};\;W_{s},\;b_{s}\right]$$ (6)

*W*_*n*_∈*R*^*n*×*m*^,*W*_*c*_∈*R*^*m*×*n*^,*b*_*n*_∈*R*^*m*^,*b*_*c*_∈*R*^*n*^ are the weight and bias that need to be optimized; *σ*_a_(•),*σ*_s_(•) are the activation function.

**Fig 3:**
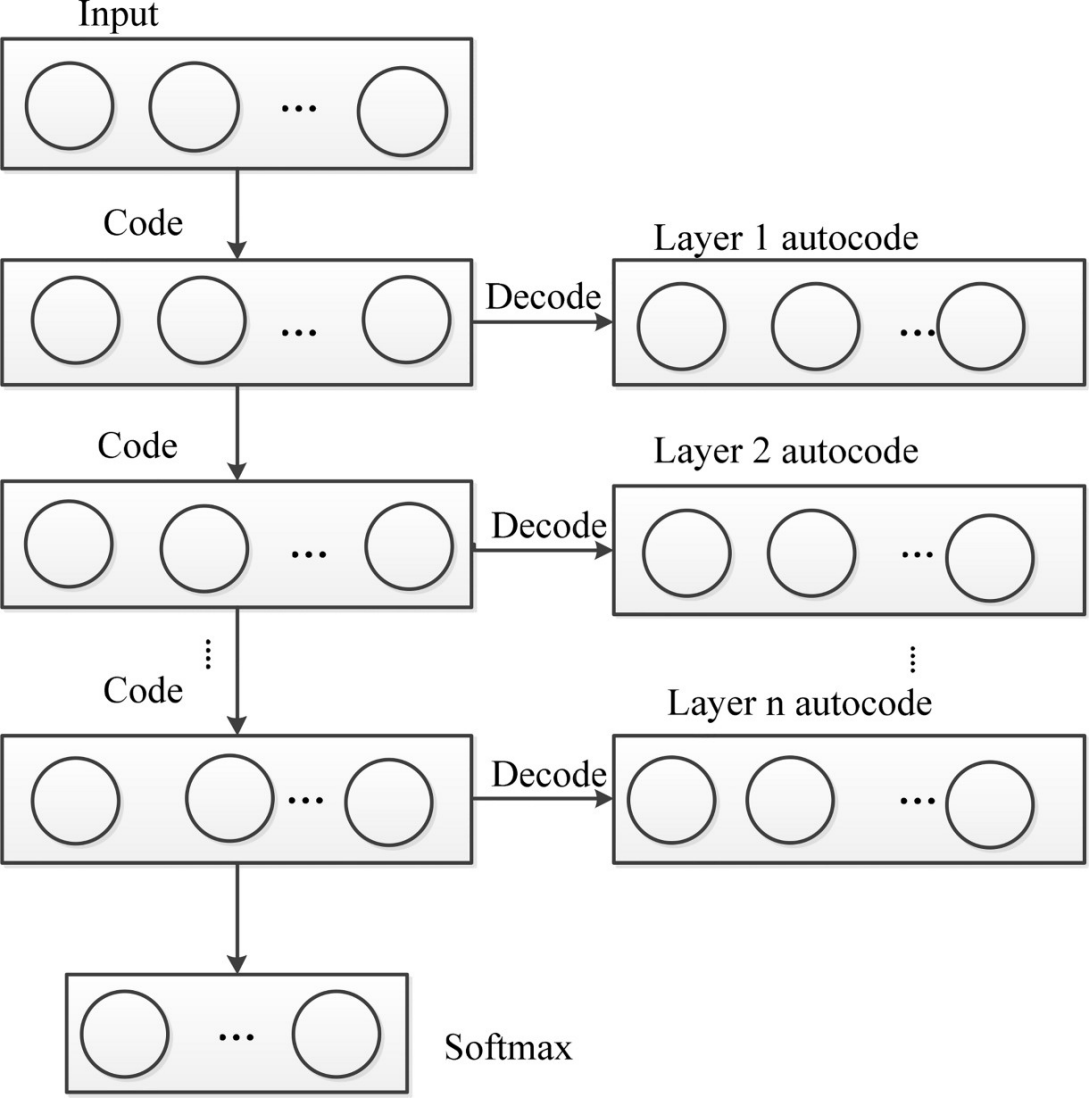
Schematic diagram of SAE structure.

The encoding layer parts of the Auto Encoder are stacked, that is, the hidden layer of the previous Auto Encoder is used as the input layer of the next Auto Encoder network. Finally, the Softmax classifier is added to the last layer of the network constitutes the Stacked Auto Encoder (SAE) [27]. SAE trains layer by layer in the order from the bottom to the top. The SAE structure diagram is shown in Fig 3.

The encoding parts of multiple Auto Encoder networks (AE) are superimposed, the input vector is received at the first layer of the AE, and sent to its hidden layer after training. The raw data is transformed layer by layer to the top layer, so that the feature of the input vector is extracted multiple times to form a more representative feature vector, and then the feature vector is classified by a Softmax classifier. This Stacked Auto Encoder (SAE) network can well extract the features in the input data, and ensure that important information in the data is not lost.

## 2 Based on GAN-SAE fault diagnosis method

**Fig 4:**
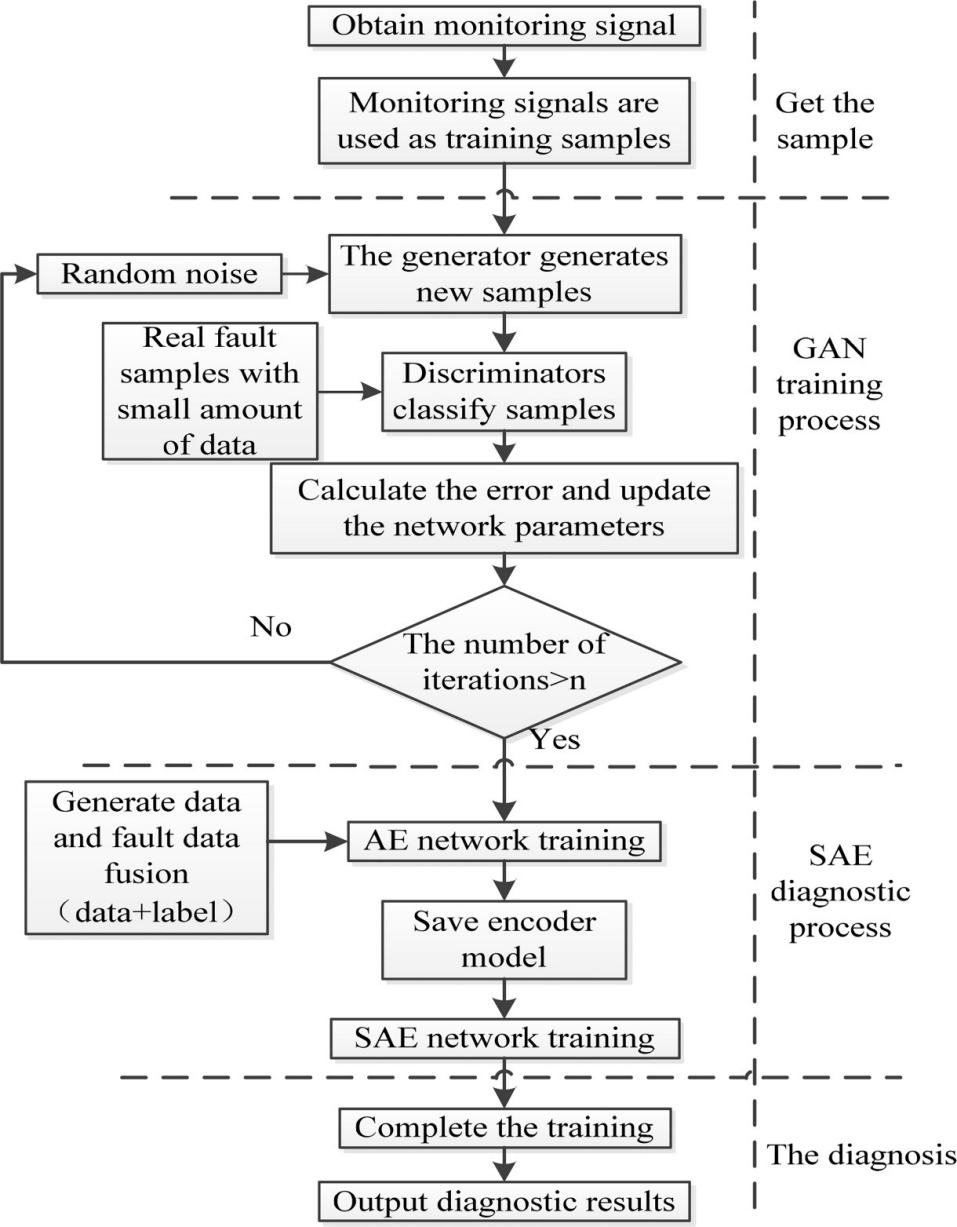
Flow chart of GAN-SAE method.

Due to the complex structure of the monitoring equipment and the harsh and variable operation environment, the massive data collected by the equipment operation monitoring system contains rich and variable fault information of mechanical equipment components [28]. Based on the data-driven fault diagnosis method, a large number of data are usually classified labeled and input to the already built deep learning network, and then the diagnosis results are got. However, in the actual production process, the equipment is in normal operation state for a long time, occasionally there are faults, resulting in a small amount of fault state data in a large number of monitoring data, then leading to data imbalance in the actual operation data set, which makes it very difficult for fault diagnosis based on data-driven methods. Since GAN is a new type of deep learning network model with strong data generation ability according to Fig 1, this paper intends to use GAN, sending random Gaussian noise variable Z into generator model G to generate a batch of new samples, which will be input into discriminator D together with real fault data samples for judgment. The generator network parameters will be updated according to the gradient of loss function. The generated samples are closer to the real fault data samples after iteration, and the loss function is formed according to the output of the discriminator. In this way, we can mine the features of data, generate data with the same feature distribution, make up for the data imbalance of sample, and improve the classification accuracy of the classifier. SAE can complete the task of dimensionality reduction and feature extraction, which can effectively avoid local optimum and improve the convergence speed of network. Based on this idea, this paper designs the fault diagnosis method integrating GAN with SAE, which is called GAN-SAE method, to diagnose the high-speed electrically driven feed pump. The flowchart of this method is shown as Fig 4.

The steps of the whole process can be summarized as follows: Firstly, obtain the detected signals during monitoring process, without feature extraction, take the detected signals directly as training samples. Secondly, build GAN network, input the training samples into the GAN network, and complete training for *n* times, until reaching the set goal, get the pseudo-samples generated by GAN combined with the real data to form a new data sample and input into the SAE network. Finally, complete the training and output the diagnosis result.

## 3 Experiment and analysis

### 3.1 Data acquisition system of electrically driven feed pump

**Fig 5:**
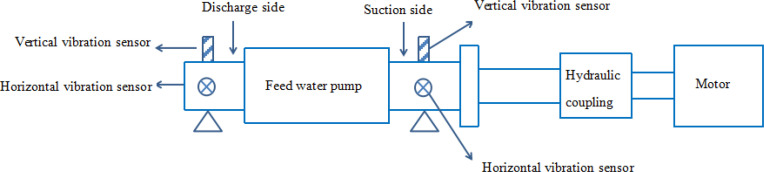
Schematic diagram of data collection system for electrically driven feed pump.

**Fig 6:**
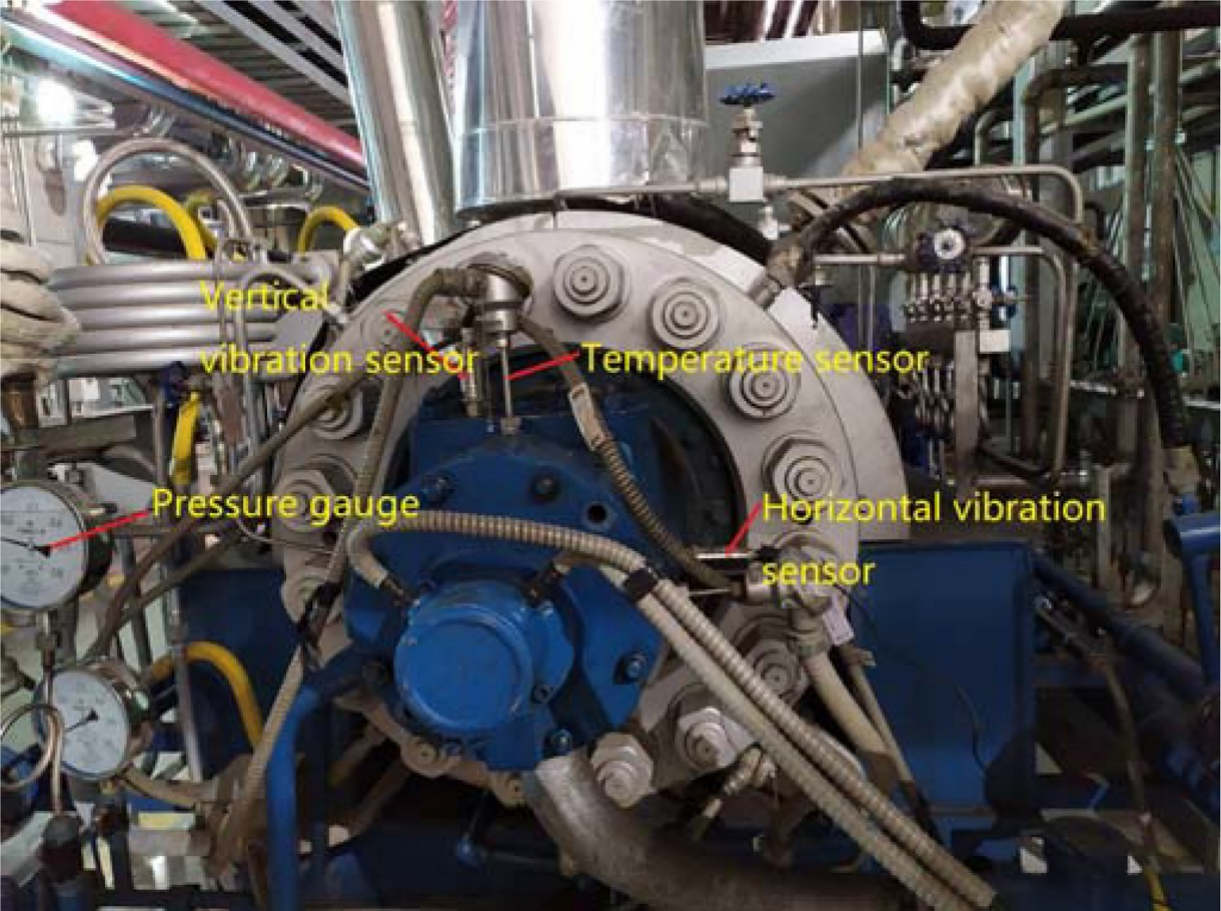
Diagram of local sensor layout.

The electrically driven feed pump unit is mainly composed of feed pump, hydraulic coupling and motor. As the main driving component, the motor drives the feed pump through the hydraulic coupling. The schematic diagram of the data acquisition system of the electrically driven feed pump shown in Fig 5. During the production process, many parameters are monitored by sensors. Such as, CD-21 vibration sensor is used to monitor the horizontal and vertical vibration of the bearings at the suction side and discharge side, FR1312 sensor to monitor the rotation speed of feed pump, PT100 temperature sensor to monitor the bearing temperature, YN100 pressure sensor to monitor the pressure of the feed pump, AVMN flowmeter to monitor water flow of feed pump and etc parameters. The diagram of local sensor distribution in Fig 6, sensors that lay at the site can be seen from the end face of feed pump, such as vibration sensors, temperature sensors and pressure sensors. According to JB/T-8097-1999, which is the national machinery industry standard, the fault diagnosis experiment was carried out for feed pump vibration.

### 3.2 Prepare experimental data

**Fig 7:**
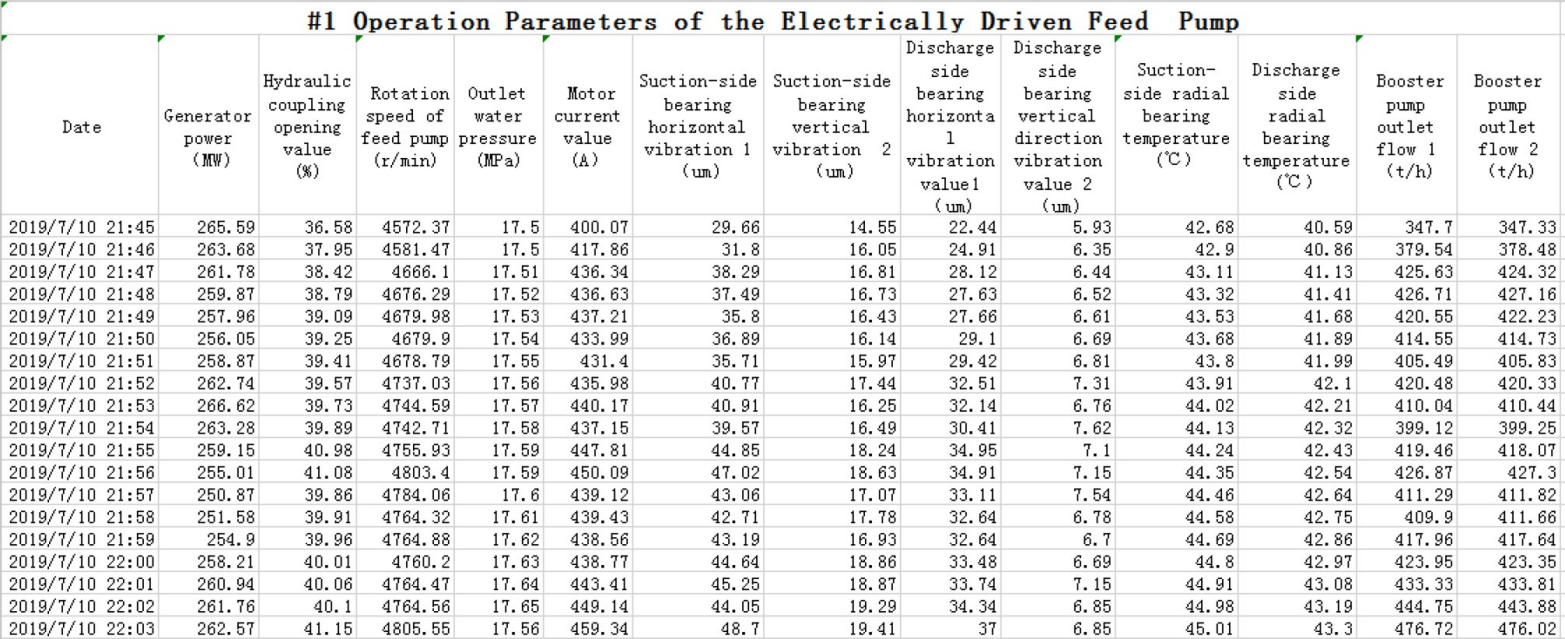
Collected field data screenshot.

The experimental data come from the monitoring data of the electrically driven feed pump in one power utility. Generally, the running speed of the electrically driven feed pump is in the range of 3000r/min to 5600r/min. The failure rate of feed pump is relatively small when it runs in low speed. the measured data are not enough to be used as the input of pump fault diagnosis, so the data are excluded in the experiment when its running speed is less than 3000r/min. In the experiment, the monitored data for a period of time after feed pump operation are selected to generate diagnosis result, which include the normal operation data of the feed pump before the fault occurs, and the fault data (such as the vibration fault), and the operation data after troubleshooting (reducing vibration by field dynamic balance treatment of electrically driven feed pump). Experimental data consist 32,000 items, including 13 variables, such as generator power, hydraulic coupling opening of feed pump, rotating speed of feed pump, outlet feed-water pressure, current, horizontal vibration value of suction side bearing, vertical vibration value of suction side bearing, horizontal vibration value of discharge side bearing, vertical vibration value of discharge side bearing, radial temperature of suction side bearing, radial bearing temperature of discharge side bearing, as well as outlet flow rate 1 of booster pump and outlet flow rate 2 of booster pump. The part of the captured field data is shown in Fig 7.

#### 3.2.1 Data processing based on GAN

Due to the large numerical differences in collected data in the field with different types of variables, in order to reduce the calculation error caused by the differences of data, to ensure the original data structure is relatively unchanged, all variables are normalized [29]. At first, the data used in deep learning usually needs to be input in the range of 0 ~ 1. Otherwise, the weight, adder calculation and final classification results will be affected by different orders of magnitude of the input value. The range method is used for normalization.

$$y=\frac{x_{i}-x_{\min}}{x_{\max}-x_{\min}}$$ (7)

Where, *x*_*i*_ is the sample value, *y* is the value after normalization, *x*_max_ and *x*_min_ are the maximum and minimum value in the sample set.

For example, the data in the first column (power of the generator) in Fig 7, normalization processing is as follows. The maximum value is 317.81029MW and the minimum value is 177.57MW from the first column, the first value 265.59MW is normalized by the above formula (8).

$$\frac{265.59‐177.57}{317.81029‐177.57}=0.627638334$$ (8)

**Fig 8:**
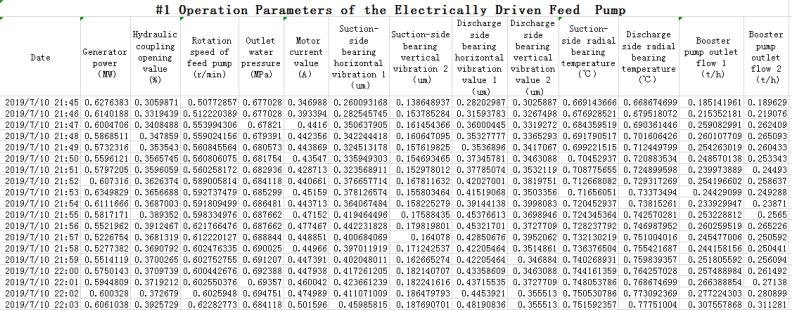
Normalized data screenshot.

Fig 8 shows a partial data screenshot after the data shown in Fig 7 is normalized by the method.

According to JB/T-8097-1999, which is the national machinery industry standard in China, during the experiments, it is a fault state if the amplitude of bearing horizontal vibration on the suction side is greater than 35um, the amplitude of bearing vertical vibration on the suction side is greater than 15um, the amplitude of bearing horizontal vibration on the discharge side is greater than 35um, or the amplitude of bearing vertical vibration on the discharge side is greater than 10um. Table 1 is the original data classification table, which gives the symbol representation in the experiment and the number of various data under various faults and normal conditions in the experiment. The symbols A represents the suction side of feed pump, and symbols B represents the discharge side of feed pump. The digital angle mark 1 represents the horizontal direction and 2 represents the vertical direction. For example, A1 indicates horizontal fault on suction side, and A1B2 indicates that horizontal fault on suction side and vertical fault on discharge side occur simultaneously. According to the statistics, the number of fault data of different categories is very different, which leads to the problem of data imbalance.

**Table 1:** Original data classification table.

	Suction-side fault (A)		Discharge side fault (B)		On both sides fault				Normal
**The fault types**	Horizontal fault	Vertical fault	Horizontal fault	Vertical fault					
**Classification**	A1	A2	B1	B2	A1B1	A1B2	A2B1	A2B2	ZC
**Classification principle**	>35	>15	>35	>10					
**Number of the fault data**	1716	203	164	454	154	133	0	836	28340

**Fig 9:**
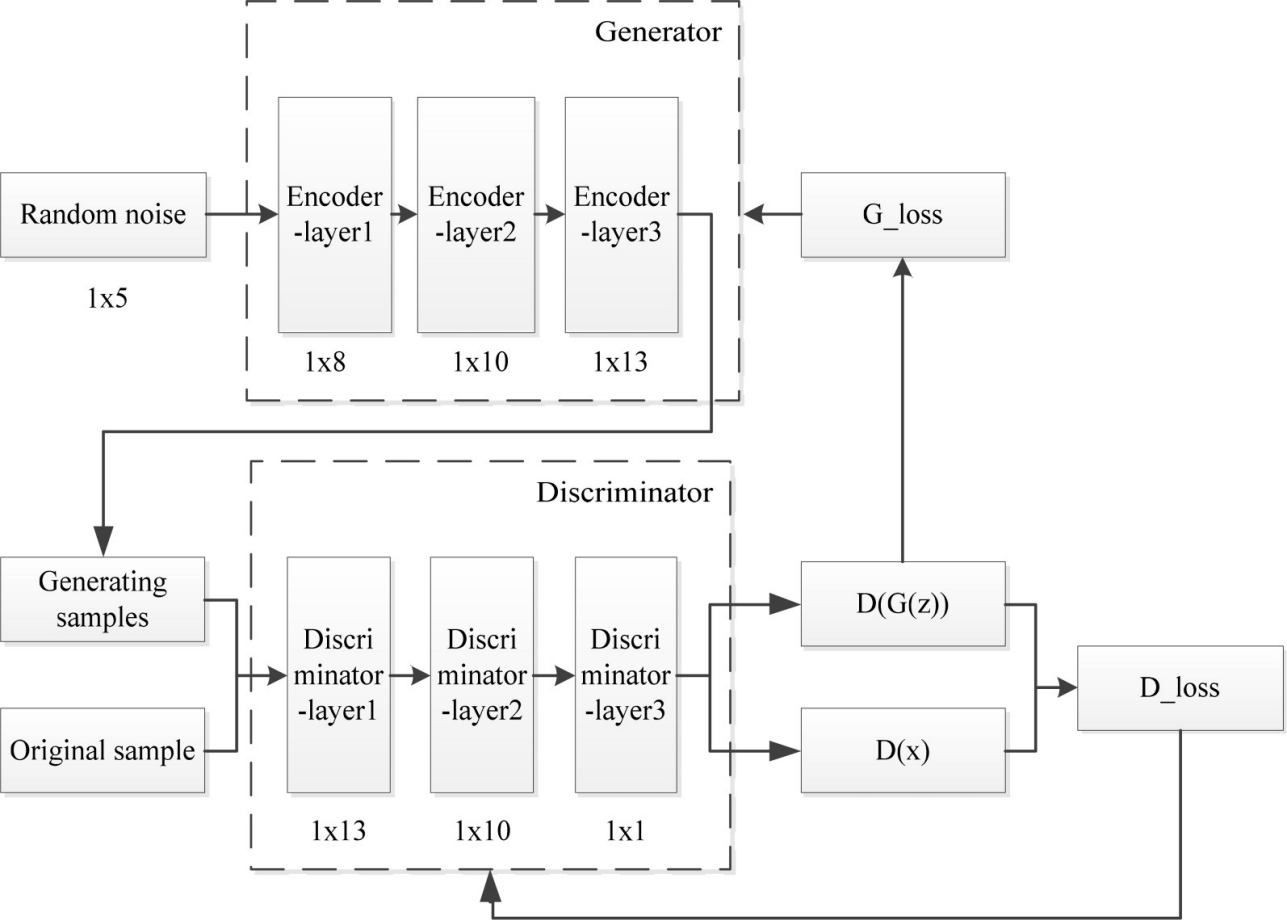
GAN structure diagram for data processing.

According to the GAN principle shown in Fig 1, th GAN structure diagram for data processing is shown in Fig 9. The network includes two parts, which are generator part and discriminator one. The generator part includes three encoder layer. The first encoder layer has 8 nodes, the second encoder layer 10 nodes, and the third encoder layer 13 nodes. Additionally, there are three discriminators in the discriminator part. The number of nodes in each layer is 13, 10 and 1 respectively. Table 2 shows the parameter settings of each encoder and each discriminator of GAN network.

**Table 2:** Parameters of the GAN structure.

Name	Numbers of node	Activation function
**Encoder-1**	8	Sigmoid
**Encoder-2**	10	Sigmoid
**Encoder-3**	13	Sigmoid
**Discriminator-1**	13	Sigmoid
**Discriminator-2**	10	Sigmoid
**Discriminator-3**	1	Sigmoid

**Fig 10:**
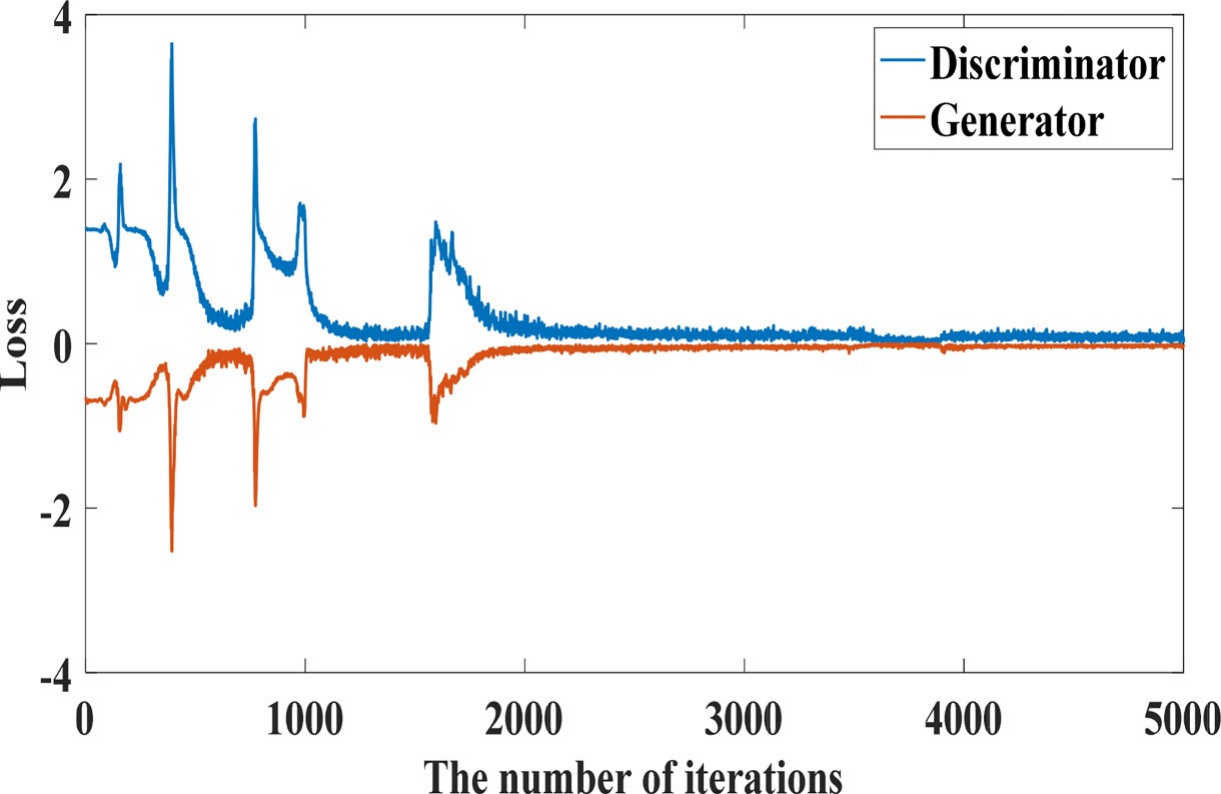
Change of GAN loss function.

After initializing the network structure and parameters, random noise is input into the generator network composed of 3-layer encoder as shown in Fig 9, and a batch of samples are generated. Next steps are to combine the generated samples with the original fault data samples and input them into the discrimination network composed of 3-layer discriminators. After training, the new generated data have a similar distribution to the original fault data, and the amount of data is compensated, so that the amount of each fault data is relatively balanced, and the number of various state data shown in Table 3 is obtained. The output of the discriminator forms the generator loss function and the discriminator loss function, as shown in Fig 10. The generator network parameters and the discriminator network parameters are updated according to the gradient of the loss function, so that the generated samples output by the generator are closer to the real fault data samples. Table 4 is the loss function change table in the iteration process. After 5000 iterations, the generator loss function becomes 0.00106, as shown in Fig 10. The loss function tends to be close to 0, indicating that the data generated by GAN has similar distribution to the actual fault data, and the generated sample data can be used as the input of fault diagnosis.

**Table 3:** Classification tables after data generation table.

	Suction-side fault (A)		Discharge side fault (B)		On both sides fault				Normal
**The fault types**	Horizontal fault	Vertical fault	Horizontal fault	Vertical fault					
**Classification**	A1	A2	B1	B2	A1B1	A1B2	A2B1	A2B2	ZC
**Classification principle**	>35	>15	>35	>10					
**Number of the fault data**	572	400	400	454	400	400	0	418	409

**Table 4:** Changes in the loss function during iteration.

Numbers of iteration	D Loss function	G Loss function
1000	1.59378	-0.87585
2000	0.34549	-0.09859
3000	0.17131	-0.05585
4000	0.11510	-0.04884
5000	0.01058	-0.00106

Lastly, remove the A2B1 type without data, combine the generated fault data with the real data, label the 7 types of fault data and the normal data respectively, and then it can be used as the sample data of the fault diagnosis system of the electrically driven feed pump.

### 3.3 Fault diagnosis experiments

#### 3.3.1 Fault diagnosis based on SAE

**Fig 11:**
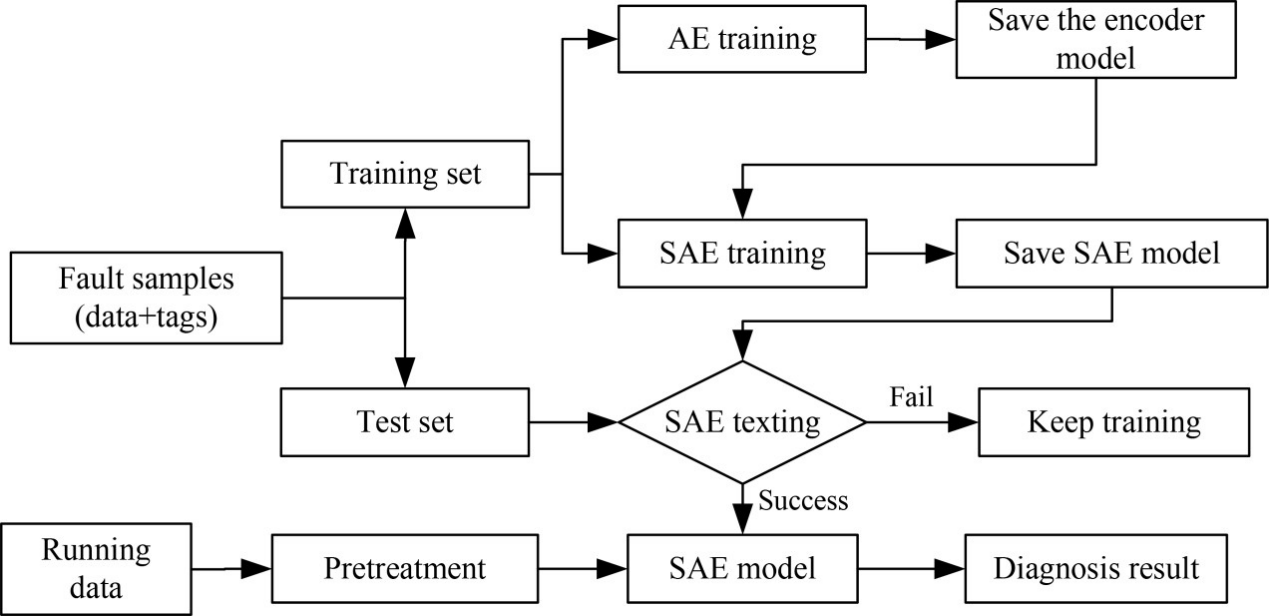
Structure of SAE diagnostic program.

The original fault data set collected in the field are used as the input of SAE diagnosis method. Two-thirds of the original fault data set will be used as the training set and the remaining one third as the test set. Using SAE method to construct a multi-layer deep learning network. The structure of the fault diagnosis program is shown in Fig 11.

Program flowchart is divided into two parts. First part is the model training process. The fault samples are preprocessed, a data set consisting of the running data and the labels corresponding to the fault types is formed. Two thirds of the data set were used as training sets and the remaining one third as test sets. The unsupervised training process of AE network only needs to run the training set to obtain an encoder, AE network can well complete the feature extraction task and save the encoder. The SAE network belongs to supervised training, whose data set consisting of running data and tags corresponding to its fault type. After reading the encoders saved in AE network, adding the category layer of Softmax as a classifier, the SAE network model can be saved by supervised training. Then the SAE network model is tested with the test set until the test model is successful. The second part is the process of fault diagnosis using SAE network model. In the process of fault diagnosis, after preprocessing the data collected in the field, the SAE model is called as the running data for fault diagnosis, and the label vector corresponding to the fault type is output to achieve the purpose of fault diagnosis. In order to reduce the influence of random factors on the diagnosis, the experiment was repeated 15 times. The SAE method has an average diagnostic accuracy of 94.9% and a maximum diagnostic accuracy of 96.5% for vibration-induced faults.

#### 3.3.2 Fault diagnosis based on GAN-SAE

**Fig 12:**
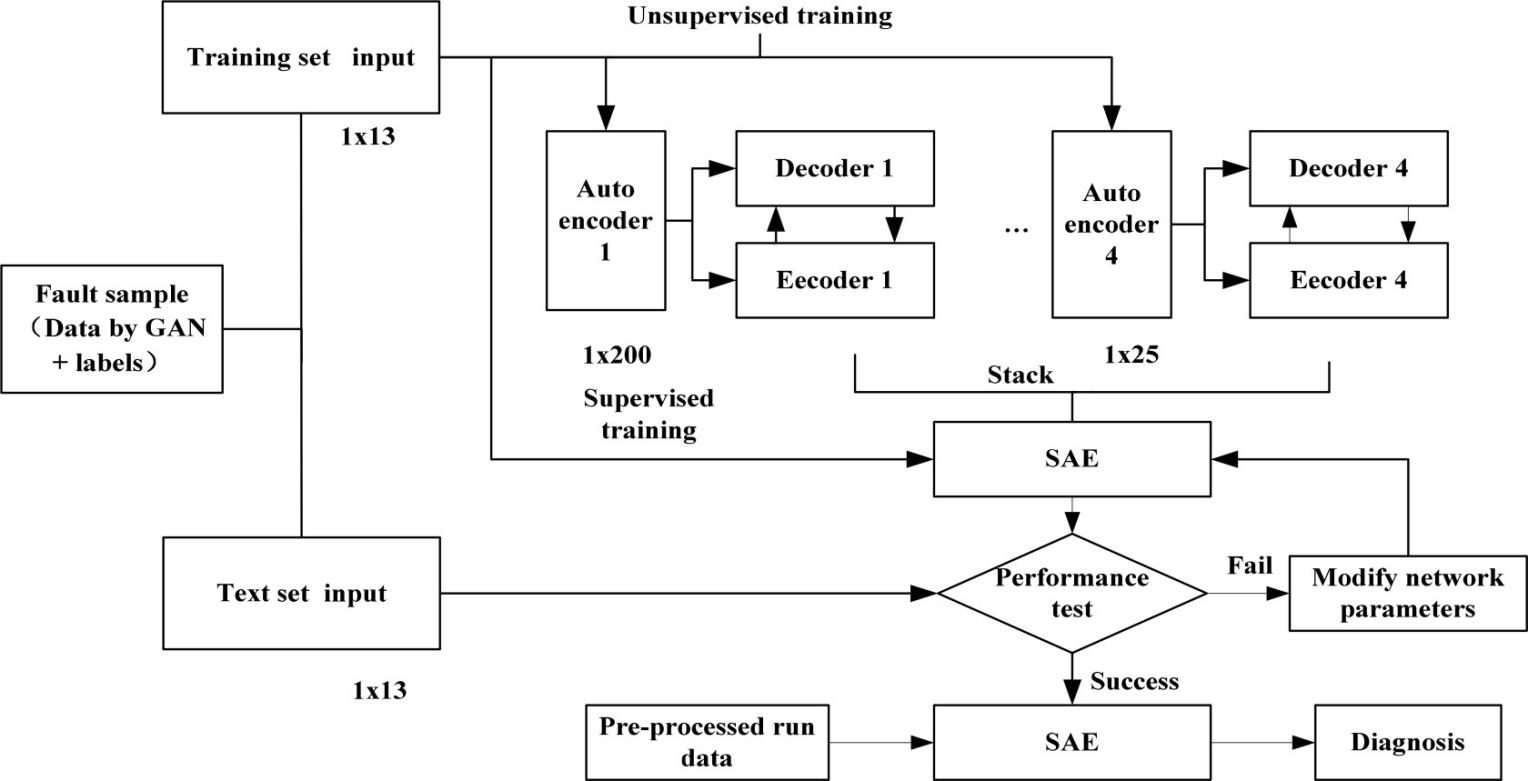
GAN-SAE network structure.

According to Fig 4, the network structure diagram of GAN-SAE method is built as Fig 12. In fault diagnosis of the GAN-SAE method, fault data is collected on site and fault sample data set is generated by GAN network, they are taken together as the input of the method, and two-thirds of the data set is also taken as the training set. It is similar to SAE method for training. The number of nodes in the network is shown in Table 5. There are 6 layers in the network, including one input layer, four encoder layers and one output layer. The number of neuron nodes in each layer is 13, 200, 100, 50, 25 and 8 respectively. GAN-SAE network model is obtained after training. In the process of fault diagnosis, after preprocessing the data collected in the field, the experiment is also repeated for 15 times as the running data. The saved GAN-SAE model is used for fault diagnosis, and the label vector corresponding to the fault type is output. The diagnosis is shown in Fig 12. The GAN-SAE method has an average diagnostic accuracy of 98.16% and a maximum diagnostic accuracy of 98.89% for vibration-induced faults.

**Table 5:** GAN-SAE network settings at each layer.

Name	Numbers of node	Activation function
**Input layer**	13	Leaky_relu
**Encoder-layer1**	200	Leaky_relu
**Encoder-layer2**	100	Leaky_relu
**Encoder-layer3**	50	Leaky_relu
**Encoder-layer4**	25	Leaky_relu
**Softmax layer**	8	Softmax

#### 3.3.3 Comparison of diagnosis results

**Fig 13:**
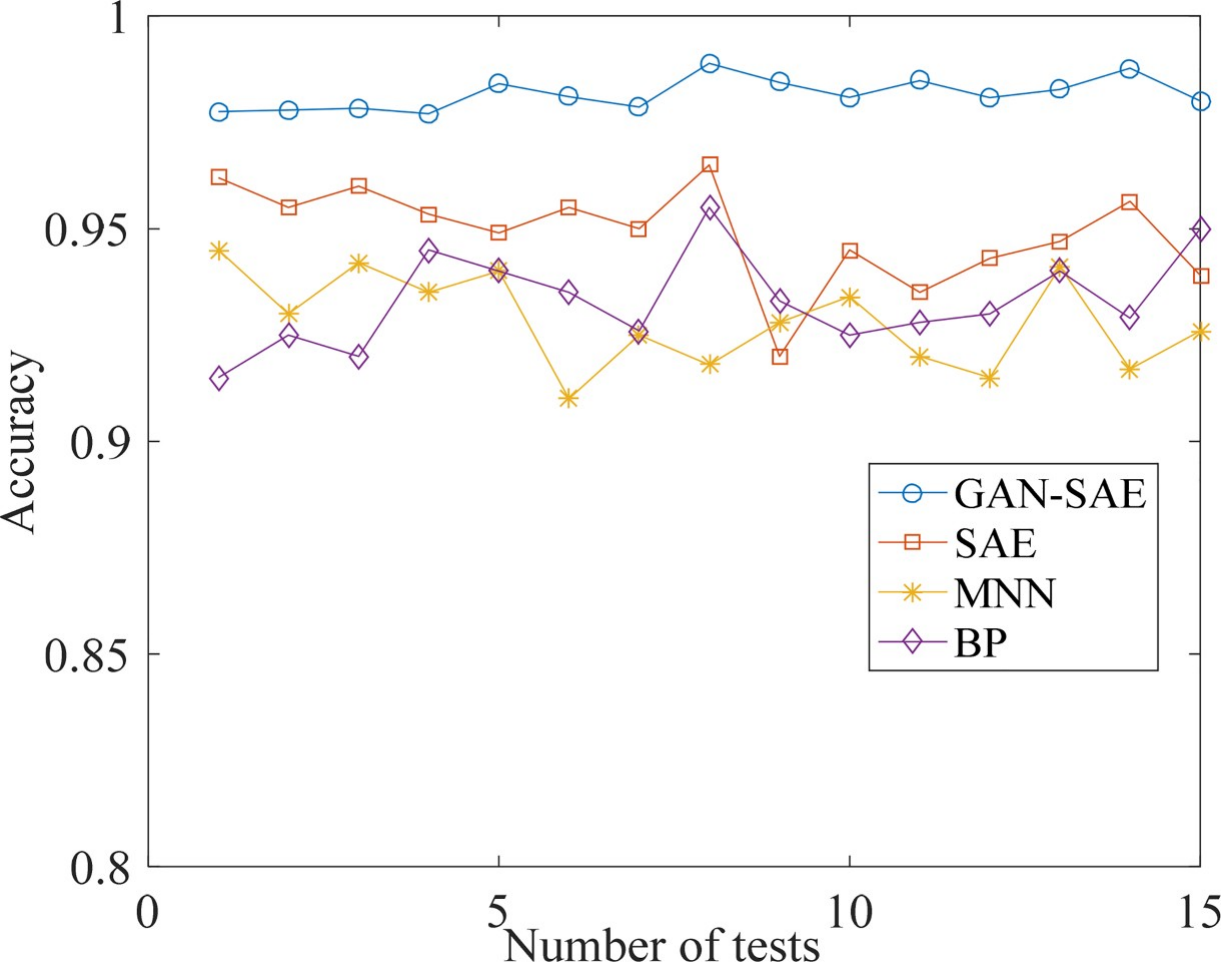
Diagnostic accuracy of the 15 trials.

In this experiment, compared with diagnostic accuracy for SAE method, GAN-SAE method, and also include other two common fault diagnosis methods: back propagation neural networks (BP) and multi-hidden layer neural networks(MNN). For BP, the number of neurons in each layer is 13, 20, 20 and 8. And for MNN, the number of neurons in each layer is configured as 13, 200, 100, 50, 25 and 8 respectively. After calculating the average accuracy (15 times) for the four methods, we got the mean and maximum accuracy for each diagnosis, and the standard deviation can be calculated. All the results are shown in Table 6. Standard deviation reflects the dispersion degree of diagnostic results. Smaller standard deviation means smaller fluctuations, which proves more stable results can be obtained by that method. Fig 13 shows the accuracy curves of the methods: GAN-SAE, SAE, BP and MNN respectively.

**Table 6:** Comparison diagnostic accuracy of different methods.

Diagnostic method	Mean diagnostic accuracy	Maximum diagnostic accuracy	Standard deviation
**GAN-SAE**	98.16%	98.89%	0.3%
**SAE**	94.9%	96.5%	1.12%
**MNN**	92.8%	94.5%	1.06%
**BP**	93.3%	95.5%	1.08%

After using the GAN to generate data and using SAE method for training, the GAN-SAE method diagnostic accuracy is at a high level with an average accuracy of 98.16% with a maximum accuracy of 98.89%. Compared with the GAN-SAE method, SAE method with the original imbalance data input gets an average accuracy of 94.9% with a maximum diagnostic accuracy of 96.5% after 15 times training, and its standard deviation is higher than the GAN-SAE method. The average accuracy of MNN method was 92.8%, the maximum accuracy was 94.5% and the average accuracy of BP method was 93.3%, the maximum accuracy was 95.5%. From Fig 13, the fluctuating range of GAN-SAE is smallest among the four methods, proved the smallest deviation value in Table 6. Although average diagnostic accuracy of SAE is higher than MNN and BP, but sometimes it may get worse result than these two methods.

The higher value for GAN+SAE and SAE are obtained after several repeat, also shows training plays a important role in diagnostic for these methods. By comparison, GAN-SAE method can not only achieve a high diagnostic accuracy, but also get a stable diagnosis result.

## 4. Conclusion

In order to solve the problem of low diagnosis accuracy caused by the imbalance of the input data for electrically driven feed pump, and overcome the deficiency of traditional imbalanced data processing methods, this paper proposes a fault diagnosis method of GAN-SAE by combining the advantages of GAN and SAE methods, and it is applied for fault diagnosis of electrically driven feed pump.

1. In the proposed method, the powerful data generation ability and data feature mining ability of the GAN method were firstly used to generate data with the same feature distribution to compensate for the imbalanced experimental sample data of electrically driven feed pump, thereby improving the classification accuracy of the classifier.
2. After being processed by GAN, the generated fault data was fused with the real data and used as the fault diagnosis sample data of the electrically driven feed pump. Then, the SAE method, which can complete the data dimensionality reduction and feature extraction tasks, was used for fault identification to avoid the local optima and improve the network convergence speed.
3. This method was applied for the fault diagnosis of electrically driven feed pump, and it demonstrates superior recognition results through the comparison with other fault identification methods.

In this paper, the GAN method was used to balance experimental data samples, the research on the single fault diagnosis has made some progress, but the compound fault diagnosis method for massive data has some problems such as unstable training, disappearing gradient, and mode collapse. How to solve the above problems more effectively and accurately, rurther research is needed.

## Supporting information

S1 Data (XLS)
